# Providing capacity in glaucoma care using trained and accredited optometrists: A qualitative evaluation

**DOI:** 10.1038/s41433-023-02820-5

**Published:** 2023-11-28

**Authors:** Patrick J. G. Gunn, Simon Read, Christine Dickinson, Cecilia H. Fenerty, Robert A. Harper

**Affiliations:** 1 Manchester Royal Eye Hospital, Manchester University NHS Foundation Trust, Manchester, UK; 2 Faculty of Biology, Medicine and Health, University of Manchester, Manchester, UK; 3 School of Health and Social Care, Swansea University, Swansea, SA2 8PP UK; 4 Centre for Applied Vision Research, City, University of London, London, UK

**Keywords:** Health occupations, Public health

## Abstract

**Introduction:**

The role of optometrists in glaucoma within primary and secondary care has been well described. Whilst many studies examined safety and clinical effectiveness, there is a paucity of qualitative research evaluating enablers and barriers for optometrists delivering glaucoma care. The aims of this study are to investigate qualitatively, and from a multi-stakeholder perspective whether optometric glaucoma care is accepted as an effective alternative to traditional models and what contextual factors impact upon their success.

**Methods:**

Patients were recruited from clinics at Manchester Royal Eye Hospital and nationally via a Glaucoma UK registrant database. Optometrists, ophthalmologists, and other stakeholders involved in glaucoma services were recruited via direct contact and through an optometry educational event. Interviews and focus groups were recorded and transcribed anonymously, then analysed using the framework method and NVivo 12.

**Results:**

Interviews and focus groups were conducted with 38 participants including 14 optometrists and 6 ophthalmologists (from all 4 UK nations), and 15 patients and 3 commissioners/other stakeholders. Themes emerging related to: enablers and drivers; challenges and barriers; training; laser; professional practice; the role of other health professionals; commissioning; COVID-19; and patient experience.

**Conclusion:**

Success in developing glaucoma services with optometrists and other health professionals is reliant on multi-stakeholder input, investment in technology and training, inter-professional respect and appropriate time and funding to set up and deliver services. The multi-stakeholder perspective affirms there is notable support for developing glaucoma services delivered by optometrists in primary and secondary care, with caveats around training, appropriate case selection and clinical responsibility.

## Introduction

Providing sufficient capacity for glaucoma care presents challenges. Approximately 3.54% of adults aged 40–80 worldwide have open angle glaucoma [1] and with an ever-aging population, numbers of UK cases are set to rise [2]. A report by the Healthcare Safety Investigation Branch concluded there is insufficient capacity to meet demand [3], with the pandemic subsequently escalating concerns [4, 5]. In addition to capacity challenges, there have been recent changes in glaucoma care recommendations.

The role of optometrists in glaucoma within primary and secondary care has been well described [6–8]. In 2009, the first National Institute for Health and Care Excellence (NICE) glaucoma guidance was published [9], making recommendations about organisation of care, including the role of optometrists and other eye health professionals, and appropriate qualifications to work within different levels of care. The 2017 NICE guidance stated there was a requirement for optometrists and other health professionals to have both a glaucoma-related and independent prescribing (IP) qualification to make management decisions regarding glaucoma treatment [10]. The College of Optometrists’ and Ophthalmic Practitioner Training (OPT) programme both offer 3-tiered higher qualifications system mapped to different levels of care provision [11, 12]. Although NICE and commissioning guidance documents specified the importance of additional qualifications for optometrists, questions arise whether the number and distribution of optometrists with qualifications in glaucoma is sufficient to meet demand [13]. Within the Hospital Eye Service (HES), comparative national scope of practice surveys showed glaucoma was the leading extended role (88% of all units involved in glaucoma care in 2020) [14]. Data from this survey also showed a large increase in optometrists delivering laser, with 14 units (16%) with optometrists delivering SLT in 2020, up from 1 unit (1%) in 2015 [15].

In 2022, the NICE glaucoma guidance was updated in response to the LiGHT trial [16], incorporating the recommendation to offer selective laser trabeculoplasty (SLT) as first-line treatment for newly diagnosed patients with open angle glaucoma and ocular hypertension, acknowledging the role optometrists, amongst other health professionals, may play in delivering SLT [17]. Whilst many studies have examined safety and clinical effectiveness of optometrists in glaucoma care [18–21], despite the marked increase in optometrists’ involvement, there is a paucity of qualitative research evaluating enablers and barriers for delivering care, and particularly across a multi-stakeholder opinion base.

The overall aims of this study are to investigate qualitatively, and from a multi-stakeholder perspective:

- Whether optometric glaucoma care is accepted as an effective alternative to traditional models by patients, providers, and other stakeholders.
- What contextual factors impact upon the development, outcome, and sustainability of glaucoma care by optometrists.

## Materials and methods

### Method of recruitment

Patients were recruited from glaucoma clinic attendees at Manchester Royal Eye Hospital (MREH) and nationally via a Glaucoma UK registrant database. Patients attending MREH clinics during September 2021 were approached during or following their appointment. Patients registered on the Glaucoma UK database were invited via an email link. Optometrists were invited to participate during a College of Optometrists’ glaucoma-related lecture, as well as a further purposive sample from optometrists known by the research team to be involved in glaucoma roles. Ophthalmologists and other stakeholders involved in glaucoma services were recruited via direct contact. Representation was sought from across all 4 UK home nations.

### Ethical approval and Patient and Participant Involvement (PPI)

Ethical approval was granted for this project (IRAS reference 276641). Patient and Public Involvement was sought from a MREH glaucoma patient to review the protocol, participant information sheets, and interview/focus group topic guides, as developed by the research team. Informed consent was taken from all participants.

### Inclusion and exclusion criteria

All participants needed to be over 18 years old, able to communicate in English (or have availability of an interpreter) and be able to provide informed consent.

Patients needed to attend a glaucoma clinic, clinicians had to be involved in delivering glaucoma-related care, commissioners and other stakeholders held responsibility for planning or delivering glaucoma-related care.

### Interview and focus group conduct and data analysis

Interviews and focus groups were conducted via password protected recorded video meetings and transcribed anonymously. Whilst topic guides (provided in supplementary material) were available for those conducting interviews and focus groups to prompt around defined topic areas, participants were encouraged to discuss other topics, with interviewers probing unanticipated areas raised. Data was analysed using the framework method for analysis of qualitative data [22]. Interviews and focus groups were first analysed separately, and initial themes identified. Transcripts were then analysed again where relationships and evolving, interlinked themes were identified within NVivo 12.

## Results

### Recruitment

There were 38 participants, the breakdown of which is summarised in Table 1.

**Table 1:** Summary of recruitment including: participant background, method of interview, sex and location within the UK.

				Location in UK				Sex	
	Interview/focus group	Number of participants	Total	England	Scotland	Wales	Northern Ireland	F	M
Patients	Patient focus group 1	7							
	Patient focus group 2	4	15	14	0	1	0	7	8
	Patient focus group 3	4							
Optometrists	Optometrist focus group 1	4							
	One to one interviews	10	14	8.5*	2	2.5*	1	5	9
Consultants	One to one interviews	6	6	3	1	1	1	3	3
Commissioners/other stakeholders	One to one interviews	3	3	3**	0	0	0	3	0
**Total**		**38**	**38**	**28.5**	**3**	**4.5**	**2**	**18**	**20**

*One optometrist was working in both England and Wales and so recorded as 0.5 for each country employed.

**One participant involved in developing national eye health pathways is an optometrist by profession, but given their main role was pathway development and commissioning and so recorded as commissioners/other stakeholders rather than under optometrists.

We recruited 7 patients from MREH and 8 from the Glaucoma UK database. Optometrists were employed in a variety of settings including primary care (main employment for 5 participants), secondary care (5 participants), community ophthalmology services (1 participant), and postgraduate optometry education or professional development (3 participants). Regarding commissioning and other stakeholders, one participant was involved in ophthalmology service design nationally (optometrist by profession), one was involved in commissioning in Northwest England and one in Southwest England. All Consultant Ophthalmologists were glaucoma specialists. The one-to-one interviews were 33–67 minutes and the focus groups 56–80 minutes duration.

### Themes

The following section outlines key themes emerging from analysed interview data. Associated, numbered quotations from participants can be found in Tables 2 to 5.

**Table 2:** Participant quotations regarding enablers and drivers; challenges and barriers.

**1: Enablers and Drivers**	
1.1	“I think we could do a lot to help people really quickly and easily, and then take pressure off the hospital to give the people who need the higher care the access to do that” (Primary Care Optometrist)
1.2	“And, we were always pushing the limits of what optometrists can do in a clinical situation, and it was very inspiring, and very motivating”. (Clinical Academic Optometrist)
1.3	“It is certainly nice to have the opportunity to check on patients a year later and see how they’ve faired …to see how these changes which can have long term effects pan out” (Hospital Optometrist)
1.4	“I wanted it to be more than that, I thought it could be more than that, which has led me down this path really…there’s wider opportunities for primary care optometry” (Optometrist involved in pathway development)
1.5	“We are not coping with demand, and the better you train and support community optometrists, the better it’s going to be for people. As simple as that…you need to expand the expertise”. (Consultant Ophthalmologist)
1.6	“If you’ve got a blind or going blind elderly patient who struggles to travel… You bring the care to them, not the other way round” (Primary Care Optometrist)
1.7	“The most important thing is having consultants who took us seriously and trained us properly…if they wanted us to work in their clinic in five or ten years, it was in their interests that we did it as well as possible” (Clinical Academic Optometrist)
1.8	“On a Friday afternoon once every few months the consultants come and sit with us in the community to learn what we’re doing… it’s great because they suddenly realise what pressure you’re under and why sometimes things are different… that builds a nice relationship (Primary Care Optometrist)
1.9	“The more you learn about something, the more you realise you don’t know, …the more you want to develop, which is why I see the optometrists who do things in our unit, being able to expand their skills, because they’re interested in learning and developing” (Consultant Ophthalmologist)
1.10	“I think the best thing is just interacting with students over case discussions…Seeing people excited and interested in glaucoma and realising that there’s more to it than they thought … it’s the communal sense of always learning” (Clinical Academic Optometrist)
1.11	“All the optometrists myself and other consultants, we all are stakeholders as far as the glaucoma team is concerned. The management keeps changing, but the team stays the same, you have to look after the team” (C03 Consultant Ophthalmologist)
1.12	“But now it’s essential, we wouldn’t be able to deliver glaucoma care without the hospital optometrists” (Consultant Ophthalmologist)
1.13	“And it’s really good to have people from different backgrounds, and that brings in other things, that are so important” (Consultant Ophthalmologist)
1.14	“If you get the right person, they’re a joy to have in clinic, they’re proactive, they help you…so that makes it easier for you to teach them… it’s been a really positive experience” (Consultant Ophthalmologist)
**2: Challenges and Barriers**	
2.1	“There was good, in that you got a lot of experience working pretty independently pretty quickly but it was a tricky situation, in a sense that there wasn’t obvious support for patients who really needed someone more experienced than me…it did feel quite intense at times” (Hospital Optometrist)
2.2	“The banding hasn’t shifted in the entire time. The risk that you’re taking on has increased and the qualifications you’re taking on have increased. I’m on Band 7, and I’ve been at the top of that – and it hasn’t changed since I started” (Hospital Optometrist)
2.3	“If other neighbouring units are able to offer a certain band to optometrists for providing autonomous glaucoma care and for independently seeing patients and having completed the training and doing it to a very high standard then I feel that they should be at par with optometrists in the rest of the country. I am not interested in what other people in this trust are getting paid…but I am keen that my team … is paid at the right level. So, obviously I am not popular”. (Consultant Ophthalmologist)
2.4	“Whilst we might have good control over our medical cohort, we don’t have influence on some of our AHPs who have a different line management system. So, it’s hard for us to be fully active in helping steer career progression” (Consultant Ophthalmologist)
2.5	“What that lesson taught me was that investing in colleagues, and letting them develop their own skills, follow their career interests is absolutely vital, because if you don’t then people will move on and go to where their career passion lies” (Consultant Ophthalmologist)
2.6	“They’ve not been in the hospital, their pressures are too high, and you think, I could do something to fix that without causing any risk to anyone. And all we need is just a system in place to make that happen” (Primary Care Optometrist)
2.7	“Don’t ask me what defines stable glaucoma because I haven’t defined that yet. But if one could define such a thing, get those people discharged from hospitals into the community, because they just don’t have capacity in the hospitals” (Primary Care Optometrist and Educator)
2.8	“We’ve had very specific issues that probably have always been there and we’re just revealing them and putting them into sharp focus, which is that the technology is very unreliable” (Primary Care Optometrist)
2.9	“So, some practices really struggle, one practice actually said, this isn’t worth our money, it’s not worth our time” (Consultant Ophthalmologist)
2.10	“The management don’t understand that just having the basic optometry degree, does not make you a glaucoma specialist. There is a reason why there is a whole Glaucoma Fellowship, a training programme, you need years of experience and even now, there are cases I don’t know what to do. How can somebody who has just done the basic degree be able to manage glaucoma patients?” (Consultant Ophthalmologist)
2.11	“I have got a sense that there’s a history that I am not party to in some of those localities. I think there are longstanding personality clashes that have been in place and the backlash of that is tensions that have remained”. (Commissioner and other stakeholder)

**Table 3:** Participant quotations regarding training, education and higher qualifications; level of professional practice; laser.

**3: Training, accreditation and higher qualifications**	
3.1	“The optometrists are all very helpful…when I was training there were things the optometrist knew far more about, so it was symbiotic. They help me and I help them” (Consultant Ophthalmologist)
3.2	“I don’t think it matters if it’s a consultant or an optometrist…if they’re not interested in teaching you then it’s a complete waste of time.” (Primary Care Optometrist and Educator)
3.3	“I enjoy training optometrists more than I enjoy training doctors. And the reason is they listen to you, so if you tell them to do something a particular way they will do it, they will not cut corners” (Consultant Ophthalmologist)
3.4	“I just get anxious when things are dumbed down and that there is an over-emphasis on what core skills can offer…In my mind it requires upskilling beyond entry level” (Hospital Optometrist)
3.5	“I’m not aware the optometry courses have changed to fully represent what we’re asking of optometrists in the community these days, of being primary care clinicians of the eye, and that’s definitely the way it’s moving up north of the border” (Consultant Ophthalmologist)
3.6	“Glaucoma can’t be just learnt out of a book. Or done on an academic course. It needs to be face-to-face with patients and closely supervised, and good quality clinical experience” (Clinical Academic Optometrist)
3.7	“If people are not doing things on a regular basis, they are only doing half a day or one day a week in hospital services, then the accrual rate of skills is very low…people need exposure and adequate support to be able to gain skills” (Consultant Ophthalmologist)
3.8	“It wasn’t very helpful to have an exam only based qualification that then lent itself to secondary care optometrists…this wasn’t very helpful to primary care and so this new model is rather better” (Hospital Optometrist)
3.9	“I think some optometrists thought they were a bit elitist… some optometrists underestimated them…a very unhappy situation, because nobody should turn up to an exam well below the standard that they need to be” (Clinical Academic Optometrist)
3.10	“I think actually the training provided by the new design probably outweighs the funding issues… it’s not discriminatory… which supports the whole system because if more people outside of secondary care environments can get involved, in the end we will have a larger workforce with the appropriate skills” (Hospital Optometrist)
3.11	“I went through the college diploma in ocular conditions until that was considered defunct…I then did the next two bits of the glaucoma syllabus until they were about to change it again and thought I’m too old and I’ve given up” (Primary Care Optometrist)
3.12	“All the skills and knowledge that I had at that stage were good, were sufficient, and I wasn’t getting much from the college certificate. So, it felt like a tick-box exercise… it would have been better had I done it first, at the beginning, rather than maybe 5 or 6 years later” (Hospital Optometrist)
3.13	“Nobody should feel that if they’ve been working as an expert in an area for many, many years that they have to re-demonstrate things that are self-evidently well within their competence” (Hospital Optometrist)
3.14	“I am struck, for example, by the non-medical health profession where there’s a suggestion that they should take on a new role that all of a sudden, they need lots of documentation around their training. In the end it is about a human with a certain training, certain skills” (Hospital Optometrist)
3.15	“We don’t know how …who out there has got these qualifications? I think there are some cases where there are optometrists with those qualifications, but because of contracting laws we can’t necessarily work with them which is frustrating” (Optometrist involved in commissioning)
**4: Level of professional practice**	
4.1	“So, I think it was always clear to me that I wanted optometrists not just to only be managing minor cases or lower risk cases” (Consultant Ophthalmologist)”
4.2	“But I am very pleased with what we have achieved because they work at the level of a senior trainee in ophthalmology, in glaucoma, in the glaucoma clinics or … and the plan is to get them to the level of a fellow in glaucoma” (Consultant Ophthalmologist)
4.3	“Don’t ask me what defines stable glaucoma because I haven’t defined that yet. But if one could define such a thing, get those people discharged from hospitals into the community, because they just don’t have capacity in the hospitals, the same problem everybody has” (Primary Care Optometrist, educator)
4.4	So, I think it’s very, very important to have an excellent relationship, but I think it’s important also to know what the Optometrist can see and cannot see and what the responsibilities they, if they want to see more, then they have to take responsibility for it. It can’t be under our responsibility” (Consultant Ophthalmologist)
**5: Laser**	
5.1	“Well, it looks as though we want to move towards being able to undertake more laser, so if that’s the case, who’s going to be able to do that? Well, we don’t necessarily have more doctors, but our optometrists would have enough skills, because they’re very good at assessing the patients, so why wouldn’t they be able to do that?” (Consultant Ophthalmologist)
5.2	“Having optometrists who are a more regular workforce is very helpful. And optometrists are very skilled in doing things more than they probably realise, they do a lot of gonioscopy and so doing a SLT laser, is something which is easily within the reach of optometrists who are trained adequately” (Consultant Ophthalmologist)
5.3	“I think if an optometrist has been adequately trained and has acquired the necessary skills, then there should be no reason why they can’t perform laser in any setting where they are. And obviously there are laser protocols, and safety issues which need to be adhered to as with anyone who uses a laser machine” (Consultant Ophthalmologist)
5.4	“I feel much more able to counsel in a supportive way from the understanding of seeing how patients cope going through the procedures and the aftereffects of the procedures.” (Hospital Optometrist)
5.5	“I am struggling logistically with how I might achieve that here without upsetting people in terms of being aggressive, or overly assertive in how I were to push it…having a great working relationship with your multi-disciplinary ophthalmology colleagues is really important. And every so often I do lightly bang my fist on the table about certain things, but there are more of them than there are of me, and so I get sort of out voted” (Hospital Optometrist)

**Table 4:** Participant quotations regarding the role of other non-medical eye health professionals; commissioning, development of local and national pathways; COVID 19; looking to the future.

**6: Role of other non-medical eye health professionals**	
6.1	“So I think it’s a little bit about who your personnel are locally… I’ve got colleagues who’ve had amazing nurse practitioners, and I’ve had colleagues with fantastic orthoptists” (Consultant Ophthalmologist)
6.2	“If you think about an ST 5 in ophthalmology who’s spent five years circling through the various subspecialties, our glaucoma specialist nurse has basically spent five years just doing glaucoma. So, she is, in many ways, more qualified to be doing a lot of these things than the medical staff” (Consultant Ophthalmologist)
6.3	“So, I think the personalities, and the aptitude and application that they can show is probably the critical thing” (Consultant Ophthalmologist)
6.4	“I think the jump between orthoptics to glaucoma is much larger than optometry, because they haven’t got the skills in their training to use slit lamp and other things like that, which are very difficult” (Consultant Ophthalmologist)
6.5	“So, there is a bit of competition among the different professional groups. But I think we have to overcome that. I am very keen that people are paid for what they do” (Consultant Ophthalmologist)
**7: Commissioning, development of local and national pathways**	
7.1	“We need to look to optometrists to deliver what they are able to deliver. They should be the primary care eye service in effect because that can’t be the GP” (Commissioner)
7.2	“There’s always been a good relationship. The ophthalmology team, the community ophthalmology team and the commissioners have met…There was a time when there was a re-commissioning story and that helped enormously” (Primary Care Optometrist)
7.3	“I’m surprised when I talk to colleagues, and they don’t know who their ophthalmology commissioning Manager is …Whereas I have them on the e-mail once or twice a week asking for figures or what’s going on or can we book more patients? (Primary Care Optometrist)
7.4	“There is an optometrist who’s an optometric advisor who’s very active, is fantastic at building relationships and makes it her job to go into the Hospital and talk to the consultants. So, this person really has made it happen” (Primary Care Optometrist)
7.5	“For glaucoma care, especially the long-term monitoring and management of care rather than the front end of the pathway, it’s really critically important that the local stakeholders design their own model that works for them…It would be lovely to have a bit more of a standardised approach to reduce the unwarranted variation but there has to be local ownership for it to work” (Commissioner)
7.6	“It is exciting to be in a position where we will see the fruits of the labour and actually the impact of the long-haul, but I’m still quite nervous because the ophthalmology community are very, very political, they are very feisty” (Commissioner)
7.7	“To not know how many patients are on your waiting list with glaucoma doesn’t sit very well with me and I know that’s a national problem” (Commissioner)
**8: COVID 19**	
8.1	“You’re sitting in a group and you’re talking around designing patient centred services and but in reality, you’re sitting with the interests of your organisation at the back of your mind …in the crisis we did. Everybody was thinking about what’s in the best interests for that patient” (Commissioner)
8.2	“The conversations we are having in 2020 and 2021 post-pandemic about recovery and transformation, you know it’s only expediting something that was happening anyway… the penny has dropped that we need more primary care engagement with this problem now” (Hospital Optometrist)
8.3	“From a clinical perspective where I am… our CCG has always been quite slow on the uptake of enhanced services. It’s sad to think that it needed COVID, but it’s gone full circle with COVID coming in, so lots and lots of enhanced schemes” (Primary Care Optometrist)
8.4	“The glaucoma monitoring has come about because of COVID waiting times” (Primary Care Optometrist)
8.5	“I know we’re talking about glaucoma, but throughout COVID we were an acute eye care hub specifically. We were offering cover seven days a week for anything. It’s really pushed the boundary” (Primary Care Optometrist)
**9: Looking to the future**	
9.1	“I think the future is optometrists in the community playing a more important role in delivering not only ocular hypertension but also mild glaucoma. That’s something that we would like to see” (Consultant Ophthalmologist)
9.2	“I guess the paradigm that we all want to achieve is things like the OLGA system, whereby the Manchester optometrists are given a lot of exposure but also are very well supported to make decisions…that’s what I’d like to replicate in my unit” (Consultant Ophthalmologist)
9.3	“I think that ophthalmology has moved on…ten, twenty years ago they would be very defensive…now the penny has dropped…we need optometry to deliver more in the community…I don’t think there are many consultant colleagues who are not accepting that…From my point of view, the more the better” (Consultant Ophthalmologist)
9.4	“We could just send in the OCTs, and someone in the hospital could perhaps assess and decide whether actually they really, really need to be coming into hospital or not” (Primary Care Optometrist)
9.5	“I’d be quite happy to take on more tricky people, as long as there was a really good system of communication in place with the hospital. And I think if that evolves over time then there’s no reason why we couldn’t see an awful lot of people in the community” (Primary Care Optometrist, educator)
9.6	“I think we’re probably nearly at our limits because you can’t go to the surgical end of things. You get to the point where you can manage the patient up to a point…and I’m happy to hand over to the ophthalmologists because they do great work” (Hospital Optometrist)
9.7	“There’s no particular reason why you couldn’t do an anterior segment injection as an Optom. I’m just not certain that I particularly want to do one” (Hospital Optometrist)

**Table 5:** Participant quotations regarding patient experience.

10.1	“I’m a consultant myself in a different speciality… the consultant I only saw her once after that it is every time a different person. I don’t mind seeing anyone as long as the person knows exactly what they’re doing…now I want to see (optometrist X) only, I don’t want to see anyone else” (Patient)
10.2	“I never really knew who I am talking to be honest apart from the nurses. I always assumed that if I meet someone in a hospital it’s a doctor” (Patient)
10.3	“I think I’m in Dr X’s clinic, I’ve not seen him for years but I’m kind of not that bothered because I have been seen by his team and it’s a team thing for me rather than an individual person. I’ve had great experiences.” (Patient)
10.4	“It’s not so much the qualification or the tag, it’s whether they’re competent to do the job or not. I found mixed services from everything from the consultant to junior people” (Patient)
10.5	“I think my concern of a study like this, it might come up with that optometrists should be allowed to perform at all levels. I don’t think it should be like that. I think it should be about individual competences” (Patient)
10.6	“We already accept that the people treating us are qualified. We don’t say well show me your diplomas…Some may have just scraped through, some may have got 90%, you never know, you’ve just got to accept that the people have had the necessary training” (Patient)
10.7	“I think delay time is a significant factor. It’s the pressure of having to chase your own appointment because if you just waited for an appointment to come out at the interval that the doctor’s written…you wouldn’t get one” (Patient)
10.8	“I transferred from the university hospital because I found it so frustrating and have gone to the community clinic. There you don’t see a consultant. I imagine I see an optometrist…it’s the consultant at the end that holds up these clinics and it’s incredibly frustrating” (Patient)
10.9	“I’m getting wonderful care from somebody in a small private opticians… as far as I am concerned I’ve be happy if she was the only person I ever saw” (Patient)
10.10	“We are lucky in the village to have extremely expert optometrist…I feel much more relaxed going to see him because every time I go to an eye clinic I’m worried I’m taking up their time” (Patient)
10.11	“They may have an optometrist already, but for some reason they get sent to us rather than them and some of our patients have been sent to other practices. So nobody’s making those connections” (Primary Care Optometrist)
10.12	“If it was the optometrist that I go to for my glasses I would be very happy with that because I have every confidence in him. If it was farmed out to (names multiple optometry practice) I would run a mile” (Patient)
10.13	“Multiples in general are maybe seen as different from independents…Patients will say things to me like, *“Oh yeah, I just go and get my normal eye test done at (multiple optometry practice), but I come here when I’ve got a sore eye”…*I think there is maybe a two–tier perception, not that there is a two–tier system, a lot of the multiples see an awful lot of red–eyes” (Primary care optometrist)
10.14	“My optometrist will show me the scans, she’ll show me the thickness of my optic nerve, what it was like last time and how much worse it is now, the results of the visual field tests. Your doctor in the clinic doesn’t have time to do that” (Patient)
10.15	“(Patient A) and my experiences are so different. Someone like me would be perfectly happy to be treated in a high street optometrist…and that would work better because that leaves consultants to help people like (patient A) who need really targeted high level medical treatment” (Patient)
10.16	“If I had the same condition as (patient B), I would not be saying what I am now…I’d be very happy to see an optometrist and see the same one each time …when I go I have no problems in waiting because I always know that when it comes to my turn they listen to what I’ve got to say” (Patient)
10.17	“To be honest, I’d prefer the nurse or an optometrist. As patient C’s point, her condition is a lot more serious than mine and I absolutely see her point of view of wanting to see a consultant. In a condition like mine where it’s just tracking it. I would rather see the optometrist” (Patient)
10.18	“What the consultant gives, is that wider experience. At *(acute NHS hospital)* they have specialist glaucoma consultants. When I was at (district general hospital) they were generalist…If somebody is coming in from *(multiple optometry practice)* starting from scratch, they would perhaps not have the experience…that is why I would always want a more senior person to oversee” (Patient)
10.19	“It’s a 150 mile round trip when I go there. It would be nice if I could get a local eye pressure test. When I got to the opticians they say you’ve got to do a full eye test… the NHS won’t allow us to do just an eye pressure test” (Patient)
10.20	“The things that do not categorically need a doctor for, if you can spread those out a bit more, fundamentally I think that is better for everybody because you may pick up those changes sooner than you would if you twiddled your thumbs for 9 months plus to get to be seen in an actual hospital” (Patient)
10.21	“We shouldn’t think of (optometrists) as cheaper and a lesser being. They will be a lot better than the consultants at the things they do more often. If I had a detached retina or something, I think I would want a consultant, not an optometrist. We’re kidding ourselves if we think the consultants know when they give me a new drop whether it will work or not, they’re just going to put it in and see. An optometrist can do that perfectly well and the consultants can concentrate on the tricky things about eyes” (Patient)
10.22	“I really don’t believe that the average intelligent optometrist is incapable of prescribing eye drops. I fully expect when there are more complicated things that he’ll send people off to the consultant” (Patient)
10.23	“I think most hospitals now, most large hospitals would have sufficient scope to focus training, not so optometrists can do every possible procedure but to have specialists in each procedure and that’s when you can get the experience which I think makes all the difference” (Patient)
10.24	“In Africa there are doctors who specialise in just doing lens replacements and they do nothing else other than lens replacements and they get through an enormous number of people. The same (would be the case) again here I think. What I would like to see is an assessment” (Patient)
10.25	You’re relying on the person pointing the thing at the right place with the right strength and for long enough…it’s the sort of thing that could be fairly easily trained and is more about responsibility and carefulness than it is about medical knowledge so I’d be more than happy” (Patient)
10.26	“On one of my trips to (the hospital) where they measure the pressure throughout the day, that was handled by very good glaucoma nurses. I often think why on earth do these expensive consultants waste time measuring my pressures… The nurse and the optometrists do it perfectly well” (Patient)
10.27	“The thing that would worry me a little would be that some of the bigger firms like (lists multiple optometry practices), might have one trained person in their business and advertise it as a full service for treating glaucoma. People could cream off the bottom end, the easy stuff” (Patient)
10.28	“I’m sure one feels it probably is different empires talking to each other that’s behind this, much as the clinics would like to have their workload reduced they’re a bit sniffy about giving some of their work to a mere optometrist…This has been talked about for years and years, about getting better linkage and it doesn’t seem to happen” (Patient)
10.29	“Most people don’t want to travel an hour and a half every three months to see them. The more that can be done by the optometrist locally. The key thing that’s missing is communication” (Patient)
10.30	Whatever level of service optometrists have the capacity to provide locally, if it can be made clear to the patient…what they can reasonably expect of the optometrist, what is the limitation on what service the optometrist can offer. There’s no uncertainty. There aren’t false expectations”
10.31	“I think delay time is a significant factor. It’s the pressure of having to chase your own appointment because if you just waited for an appointment to come out at the interval that the doctor’s written on the sheet that you hand in, you wouldn’t get one. Continuity would be a real luxury. I would quite like to see the same person that looked at the results more than once” (Patient)
10.32	“I think the most important thing is that you get the treatment for whatever the problem is as quickly as possible by people who’ve had the most appropriate training and what you call them is neither here nor there. You’re getting treatment, the best treatment by the best and most appropriate people quickly and I think that’s what we all want” (Patient)

### Enablers and drivers

Optometrists were driven to work in glaucoma clinics due to clinical interest, motivation to support overburdened clinics, and professional satisfaction [1.1]. Others expressed they found it rewarding to progress their clinical skills through work in glaucoma clinics [1.2], while being able to offer continuity of care with patients was also mentioned favourably [1.3]. That said, an optometrist who later became involved in developing care pathways stated they felt let down following their initial qualification, believing they could make greater contributions [1.4].

Having optometrists delivering glaucoma-related care in the community due to demand and ease of access for patients was noted as a driver by ophthalmologists and optometrists [1.5, 1.6]. Mutual respect between professions was reported as important in successful service implementations [1.7, 1.8]. Many responsible for training optometrists, including clinical academics and ophthalmologists, viewed the process positively and considered them highly motivated and responsive to guidance [1.9, 1.10].

Within the HES, optometrists were often reported as key stakeholders within glaucoma teams [1.11]. For some ophthalmologists, optometrists were critical to the success of services, whilst others highlighted benefits of multidisciplinary teams [1.12, 1.13]. It was also noted personality and motivation was more important than professional background [1.14].

### Challenges and barriers

Clinical support was raised as an important factor for optometrists working in HES clinics, with minimal ophthalmology assistance being reflected upon negatively [2.1]. Appropriate banding within secondary care was also raised as problematic by several optometrists and ophthalmologists [2.2, 2.3]. In addition, some ophthalmologists felt frustrated they had less influence in positively steering career progression [2.4]. Staff retention was a challenge in some units, with one ophthalmologist observing the importance of providing career progression [2.5]. Many primary care optometrists felt frustrated they could do more for patients experiencing long waits [2.6, 2.7]. Technology was often reported as a barrier to success, with system links between primary-secondary care still seen as gradually evolving versus being solidly in place [2.8]. Funding for services to run in primary care were raised as a concern, particularly in relation to administration costs associated with the service [2.9].

The question of additional training beyond core competency was raised by optometrists and consultant ophthalmologists, with differing views being presented on the levels required [2.10]. It was noted tensions between different stakeholders, e.g. between primary and secondary care, or between secondary care providers, were detrimental to progress [2.11].

### Training, accreditation, and higher qualifications

Optometrists and ophthalmologists spoke positively about experiences of training [3.1, 3.2], with ophthalmologists consistently highlighting they found training optometrists rewarding [3.1, 3.3]. Many ophthalmologists and optometrists supported training beyond core competency to work in glaucoma [3.4]. There were also questions around whether UK optometry courses had adapted, reflecting the ever-increasing role optometrists play in glaucoma [3.5]. Various training models were discussed, including apprentice-style training as well as higher qualifications. The importance of clinical experience was highlighted by many [3.6, 3.7].

Regarding the College of Optometrists’ higher qualifications, similar themes emerged regarding old and new qualifications [3.8, 3.9]. Whilst the issues of cost and placements were raised about new-style qualifications, consensus suggested these were more accessible than older qualifications [3.10]. Optometrists working in glaucoma services for some time described frustrations undertaking qualifications [3.11, 3.12] and these were understood by some in secondary care and relevant to consideration of accreditation of prior experience [3.13]. Indeed, some felt that there was a lack of consistency between expectations for accreditation between medicine and optometry [3.14]. Lack of data around workforce with glaucoma-related qualifications was highlighted as a potential barrier to developing services [3.15].

### Level of professional practice

The optometrists we spoke to were working in a variety of roles and managing differing case mixes, with some managing lower risk patients, others managing complex glaucoma. An ophthalmologist who had established a glaucoma filtering service and optometry-led HES glaucoma clinic 20 years ago, noted they always wanted optometrists to manage more complex glaucoma [4.1], whilst others were keen to develop their team to reach this level [4.2]. There were many optometrists and ophthalmologists keen for patients to be seen in primary care [4.3]. However, for some ophthalmologists, it was considered optometrists should take clinical responsibility for seeing patients in primary care [4.4].

### Glaucoma laser procedures

We spoke to a mixture of clinical staff and patients about optometrists delivering glaucoma-related laser. Overall, ophthalmologists were enthusiastic about optometrists, or nurses, taking on the role [5.1, 5.2]. When it came to the matter of optometrists delivering laser in primary care, some ophthalmologists were open to the idea [5.3]. An optometrist, delivering glaucoma-related lasers in the HES, considered their training helped with other aspects of glaucoma management [5.4]. The pathway to optometrists delivering lasers was not always straightforward, with consultant support sometimes being seen as inconsistent or unavailable [5.5].

### Role of other non-medical eye health professionals

Optometrists and ophthalmologists spoke positively about other eye-health professionals including nurses and orthoptists in delivering care [6.1, 6.2]. Both patients and clinicians highlighted that personality and individual characteristics were more important than profession [6.3]. Several ophthalmologists highlighted optometrists’ existing skillset being advantageous for training [6.4]. However, it was noted by others that there were some tensions between professional groups working in glaucoma in their unit and regarding banding [6.5].

### Commissioning, development of local and national pathways

The importance of the role of optometrists in the primary eye-care pathway was highlighted by commissioners [7.1]. Relationships between commissioners, clinicians, and those involved in designing local and national pathways was highlighted by stakeholders as a priority for success [7.2]. For some, the lack of awareness around the role of local commissioners was seen as surprising, with clear communication between all parties being regarded as beneficial [7.3]. Another optometrist highlighted how sometimes having one proactive individual can facilitate progress in establishing services [7.4]. Commissioners felt local stakeholders should have a say in how pathways are designed [7.5], though some highlighted detrimental volatility in ophthalmology community relationships between primary and secondary care, and indeed between consultants in different units [7.6]. Lack of data for glaucoma waiting lists was cited as a frustration for commissioners and others involved in planning [7.7].

### COVID 19

Many respondents highlighted changes during the COVID-19 pandemic. For some this took the shape of patients’ best interests coming to the forefront [8.1]. Others considered changes during the pandemic were expediting the shift towards primary care engagement [8.2]. For example, COVID was seen to have encouraged growth in enhanced services in their area by some optometrists, pushing the boundaries of community services [8.3, 8.5]. An exacerbation of existing waiting list problems during COVID was identified as reason for the development of a monitoring service [8.4].

### Looking to the future

When asked about the future, many ophthalmologists highlighted optometrists would play an increasingly significant role in delivering both primary and secondary glaucoma care [9.1, 9.2, 9.3].

For some optometrists working in primary care, they were keen to be more involved in glaucoma, with some raising issues around data exchange and remote HES decision making [9.4]. For other optometrists, it was felt good communication with secondary care could facilitate management of more complex cases [9.5]. Some experienced optometrists in secondary care believed they had reached the limits of their progression [9.6, 9.7].

### Patient experience

Continuity of care was reported as a priority for many patients, rather than being seen by a particular professional [10.1]. Patients attending clinics were not consistently clear of the professional they were seeing, often assuming they were a doctor [10.2]. Another patient considered it was about the team versus the individual [10.3]. Competence for all clinical staff was raised as key by some patients [10.4, 10.5, 10.6], alongside access to care and waiting times [10.7, 10.8].

Some patients reported strong relationships between themselves and their primary care optometrist [10.9, 10.10]. When considering primary care, patient choice of practice was raised as important by both patients and clinicians [10.11, 10.12]. Patient perception between independent and multiple optometry practices was raised by an optometrist [10.13]. Some patients felt their primary care optometrist may spend more time discussing test findings in comparison to expectations in secondary care [10.14].

Within focus groups, patients with differing complexities of disease discussed their views on who should be treated by whom [10.15, 10.16, 10.17]. Those with advanced glaucoma sometimes described their preference to be under consultant-care [10.18]. However, this particular respondent also felt frustrated they were unable to access primary care optometry for intermittent checks on their pressure [10.19]. Many patients we spoke to seemed happy to be seen by optometrists for aspects of their glaucoma care when they felt this was low risk [10.20, 10.21].

When discussing trained optometrists prescribing glaucoma medications, patients were positive [10.22]. Regarding optometrists delivering glaucoma-related lasers, patients reported training, assessment, and volume of patients were important considerations [10.23, 10.24, 10.25]. The financial aspect of non-medical health professionals (NMPS) delivering care was raised by a few patients [10.26]. Some reported a concern or perception of privatisation of the NHS [10.27]. Communication between secondary and primary care was highlighted as an issue [10.28, 10.29, 10.30].

When questioned about the most important part of their care, patients reported reducing delays, quality and continuity of care were key factors [10.31, 10.32].

## Discussion

Optometrists provide significant capacity within glaucoma pathways, involving detection, diagnosis, monitoring and treatment [23, 24]. Despite these contributions, there has been little qualitative research on whether care by optometrists is an accepted alternative to traditional-care, and what factors impact development and sustainability of glaucoma services with optometric involvement. The results of this study show broad support for optometrists delivering glaucoma care, providing insight into multi- stakeholder opinions.

All stakeholders saw potential for expanding glaucoma-related provision in primary care, many reporting this being key to dealing with the capacity crisis. Where primary care services were already running, optometrists felt valued, highlighting the importance of good primary-secondary care communication as vital to maintain and develop services, a factor also emphasised by patients. Patients self-identifying as lower risk were more accepting of care in primary care, as well as those with good relationships with their primary care optometrist. Some patients felt they would receive greater consistency of care in primary care, but those with more complex glaucoma appeared more inclined to being seen in secondary care.

There has been previous work canvassing patient perception of optometrists delivering glaucoma care in the community [25]. However, little qualitative information was presented in this early study. Our study shows appropriate case selection and adequate practitioner training/experience are most important to patients when considering willingness to be seen in primary care. There was suggestion from some about perceived NHS privatisation if care was provided by group-based practices, with many patients preferring to see their usual optometrist. For those establishing primary care services, providing patient choice of practice may enhance patient acceptance. Good communication between primary and secondary care was referenced as an enabler across participants and as a barrier when communication failed. The use of two-way electronic communication has been described to positively benefit glaucoma referral filtering pathways [26, 27] as well as help successfully facilitate glaucoma virtual clinics [28–30]. Investment in well-functioning digital technology should therefore be prioritised, a view supported by participants in our study, and a priority for the government [31] and the NHS [32, 33]. The use of artificial intelligence is proposed to have potential to enhance capacity [34, 35] and further qualitative research about patient and clinician perception of its use would be beneficial.

Positive relationships between optometry and ophthalmology were consistently reported as crucial. For example, where ophthalmologists had invested in training and mentoring, optometrists felt more valued, appearing more committed and empowered to participate in advanced roles. Where relationships were less developed or had broken down, this scenario was a barrier to implementing successful primary care-based services. Ensuring all stakeholders have a voice when establishing services is recommended.

Within secondary care, patients experiencing optometric care spoke highly of their experiences. Given the multidisciplinary nature of HES clinics, patients were sometimes unaware of their clinician’s profession. When asked, some patients reported a preference for seeing an ophthalmologist, citing condition complexity, although such patients had often not experienced optometric care. The use of risk stratification such as the Royal College of Ophthalmologists’ and UKEGS’s joint guidance [36] could help select appropriately matched cases to clinicians. However, some patients reported a preference to see an optometrist and felt competence was about the individual not the profession. Considering patients’ preferences vary, and limitations in risk stratification approaches in advanced glaucoma [37, 38], a bespoke approach based on available local expertise and canvassing feedback from patients within individual services may enhance clinical effectiveness and patient acceptance. Further research into experiences and perceptions of patients with advanced glaucoma attending multidisciplinary services, may help guide those planning services for higher risk patients.

Recruiting and retaining optometrists within secondary care was reported as a challenge by ophthalmologists and optometrists. Banding and career progression were cited as key reasons, with some optometrists reporting case complexity had increased without any associated rise in banding. Interprofessional banding has previously been discussed by Greenwood et al., noting apparent differences in banding between professional groups [39], acknowledging differences may relate to the level of clinical practice and autonomy, in keeping with the views of clinicians in this study. The consensus view was that clinical staff should be paid for their level of clinical practice, autonomy, as well as additional responsibilities within a service, regardless of professional grouping. Service providers may need to ensure they can provide appropriately banded opportunities, recognising the increasingly highly specialised role optometrists and other health professionals have within glaucoma. Leadership development such as the Mary Seacole Programme and the advanced clinical practice (ACP) framework [40] may help support staff development and retention. However, difficulties in ACP implementation have been highlighted [41] and other factors raised by ophthalmologists as problematic to staff retention [42] are likely relevant to other NMPs. Further research in these areas would be beneficial.

The recent NICE guidance for glaucoma recommended SLT be offered as first line treatment, with implications for glaucoma services in providing capacity [17], highlighting the role of healthcare professionals meeting this demand, and stating expectations for training and supervision. Whilst the recent scope of practice of hospital optometrists [14] showed an increase in numbers of optometrists delivering SLT, greater capacity will be needed to meet NICE recommendations. Recently, Konstantakopoulou et al., conducted qualitative research exploring acceptability, enablers and barriers of optometrists delivering SLT [43], concluding an optometrist-delivered service could benefit the NHS. Whilst this work offered a useful multi-stakeholder perspective, it was limited to patients and clinicians from one hospital. The results of our study similarly found a positive perspective for optometrists delivering SLT. With regards to optometrists delivering SLT in primary care, we also noted some patients and clinicians expressed reservations. However, others were open to a primary care-based service, with caveats around appropriate training, equipment, and links to secondary care. One non-randomised UK-based study comparing clinical effectiveness of SLT by NMPs to ophthalmologists [44] showed NMPs to be safe and effective. Further studies may be helpful in providing reassurances.

There was some tension relating to higher qualifications. Some respondents felt these enhanced performance and confidence, where others considered these overly onerous, expensive, and lacked flexibility for those with significant experience. Work by Myint et al., [45] highlighted the importance of practice-based training when developing skills in glaucoma assessment. Having a range of routes to accreditation, including accommodation of prior clinical experience, may reduce obstacles, and programmes such as the OPT [12] and funded ACP apprenticeships may support this. The 2022 NICE guidance states those involved in monitoring and treating patients should have a specific qualification in glaucoma [17], and as highlighted in our study, further work evaluating the workforce with glaucoma related qualifications will help support service planners. Regarding accreditation for performing SLT, in keeping with Konstantakopoulou et al., [43], our study emphasises the importance of gonioscopy, laser safety and patient counselling, rather than a specific qualification. Translimbal direct selective laser trabeculoplasty [46], a newer technique currently being evaluated [47], may lead to simplified training, and support an increased workforce for delivering laser.

There has been some qualitative research surrounding other alternative models of glaucoma care, including glaucoma virtual clinics, but none relate specifically to NMPs [48–50]. Baker et al., evaluated multi-stakeholder perspectives of the Manchester Glaucoma Enhanced Referral Service (GERS), concluding optometrists can deliver a high-quality service, acceptable to patients, commissioners, ophthalmologists, and other optometrists. Although Baker’s study was about an enhanced referral service, their findings align with ours. The significant role nurses, orthoptists, and other health professionals play in glaucoma services was highlighted by participants in our study and has been described elsewhere [2, 51, 52]. Whilst the focus of our study was on optometrists, given the scale of their numbers working in both primary and secondary care, further explorative work regarding broader NMP groups in delivering glaucoma care would be beneficial.

Our study is the first to secure multi-stakeholder perspectives from all four UK home countries on the role of optometrists delivering glaucoma care. Whilst we received a perspective from patients attending different models of care in England and Wales, most had their care delivered in secondary care, and further insight from those receiving follow up in primary care would be beneficial, as well as seeking a broader perspective of patients in Scotland and Northern Ireland. Despite this limitation, our study affords a broad multi-stakeholder perspective on the role of optometrists in delivering glaucoma care, with collaborative working, trust and keeping patients at the centre of care all being key priorities. Having an improved understanding of how to successfully engage optometrists within glaucoma services should enable service leads, commissioners, and other stakeholders to work towards the shared goal of maximising capacity and quality within glaucoma services.

## Summary

### What was known before

- Optometrists contribute significantly to providing capacity in glaucoma care.
- There is multi-stakeholder support for enhanced referral services delivered by optometrists.
- There is evidence to support the role of optometrists delivering SLT laser in a large tertiary hospital.

### What this study adds

- There is notable support from patients, ophthalmologists, optometrists, commissioners, and other stakeholders for developing glaucoma services in primary care, with caveats around training, appropriate case selection and clinical responsibility.
- Success in developing glaucoma services with optometrists and other health professionals is reliant on multi-stakeholder input, investment in technology and training, inter-professional respect and appropriate time and funding to set up and deliver services.
- A broader understanding of the viewpoint of patients, clinicians and other stakeholders of the role optometrists could play in delivering SLT laser.

### Supplementary information

Supplementary Material

## Data Availability

The data that supports the findings of this study are available from Manchester University NHS Foundation Trust, but restrictions apply to the availability of the data due to participant consent. The data may, however, be available from the authors upon reasonable request and with the permission of Manchester University NHS Foundation Trust.
