# Supplementary material for: Providing capacity in glaucoma care using trained and accredited optometrists: A qualitative evaluation
Source: Eye (Lond). 2023 Nov 28;38(5):994–1004. doi: 10.1038/s41433-023-02820-5 (PMC10966092; doi:10.1038/s41433-023-02820-5)
Supplement: Supplementary file 1 — Supplementary Material [file 41433_2023_2820_MOESM1_ESM.docx]

**Capacity and the role of glaucoma optometrists: a qualitative study**

**INTERVIEW SCHEDULE – Patients**

This interview will involve questions about your experiences and encounters of optometrists working within glaucoma care. We may also talk about your feelings or experiences with doctors or other health professionals in the past.

The schedule below outlines the proposed questions that may be asked during the 30 to 45 minute interview. However, this is an outline and deviations may be made from the schedule to develop the questioning if the conversation offers information that may benefit the research study.

**Objectives:**

1. Whether with further training and accreditation, optometrists can provide a high-quality glaucoma service that benefits the overall eye care pathway.
2. Whether optometric glaucoma care is accepted as an effective alternative to traditional models of care by patients, providers and other stakeholders.
3. What contextual factors impact the development, outcome and sustainability of glaucoma care by optometrists.

**At the beginning of the interview:**

- Explain the purpose of the project.
- Explain the format of the interview: length, topics to be covered, flexibility in structure.
- Highlight the importance of participants’ responses: they are the experts.
- Reiterate consent procedures and offer opportunity to ask any further questions.

**Guide for questions:**

- How did you come to be seen in the glaucoma clinic?
  - How long have you been coming to the clinic for?
  - Do you know what your diagnosis is?
  - How long have you been getting your care delivered by an optometrist?
  - Have you previously had your care delivered by other professionals (e.g. doctors, nurses, orthoptists, technicians)
- Are you always aware of the profession of the member of staff who conducts your glaucoma appointment?
- Do you know what an optometrist is and what their role involves?
  - What training do you think optometrists working in your clinic will have had?
  - When changes to your treatment or management plan are made, are these done independently by the optometrist or are other members of the team consulted with?
  - Have you had medications prescribed by an optometrist before?
- How do you feel about having an optometrist delivering your glaucoma care compared to other health professionals?
- Do you have a preference about what profession your clinician in the glaucoma clinic is?
- Have you had any treatment such as a glaucoma laser procedure delivered by an optometrist?
  - If so, how confident were you with the care you were given?
  - If not, how would you feel about having a glaucoma laser procedure performed by an optometrist?
- Are there any particular aspects of having your care delivered by an optometrist you like?
- Are there any particular aspects of having your care delivered by an optometrist you don’t like?
- How confident are you in the care provided by the optometrist?
- Do you trust the optometrist delivering your care?
- What do you think the benefit to your NHS trust is, by having optometrists deliver your care?
- How do you feel about not being seen by the consultant that supervises your care in clinic?
- Overall, would you prefer to be seen in your current glaucoma clinic where you may be seen by an optometrist or another clinic type where you would be seen by another health professional?
- If you could change one thing about your glaucoma care, what would it be?

**INTERVIEW SCHEDULE – Clinicians and other stakeholders**

This interview will involve questions about the experiences, concerns and encounters of optometrists or other stakeholders working with optometrists in glaucoma care. We may also talk about feelings of experiences working with different types of health professionals in the past.

The schedule below outlines the proposed questions that will be asked during the 30 to 45 minute interview. However, this is an outline and deviations may be made from the schedule to develop the questioning if the conversation offers information that may benefit the research study.

**Objectives:**

1. Whether with further training and accreditation, optometrists can provide a high-quality glaucoma service that benefits the overall eye care pathway.
2. Whether optometric glaucoma care is accepted as an effective alternative to traditional models of care by patients, providers and other stakeholders.
3. What contextual factors impact the development, outcome and sustainability of glaucoma care by optometrists.

**At the beginning of the interview:**

- Explain the purpose of the project.
- Explain the format of the interview: length, topics to be covered, flexibility in structure.
- Highlight the importance of participants’ responses: they are the experts.

- Reiterate consent procedures and offer opportunity to ask any further questions.

**Guide for questions:**

- How did you come to work within glaucoma care?
  - What is your profession?
  - How long have you been working in this clinic for?
  - Have you ever worked in different glaucoma clinics (prompt for further information if appropriate)?
  - What involvement do optometrists have in your glaucoma clinic?
- What training and support have you/optometrists working in your clinic received?
  - Training
    - Has training been formal, face to face, distance learning etc?
    - Are there any specific qualifications you have acquired for the role (if specific, has the employer funded this)?
    - Do you/optometrists working in the clinic receive ongoing training and education?
  - Support
    - Are there clear guidelines or processes in place in your clinic?
    - Do you/the optometrists working in your clinic feel adequately supported in the role?
    - If assistance is required for patients in an emergency, is help easily accessed? And which member of the team do you normally ask?
    - How do you feel other members of the team view you / optometrists working in the glaucoma clinic?
- Do you/optometrists in your place of work deliver glaucoma care?
  - If yes
    - How/when were these clinics set up?
    - What other staff groups work in these clinics?
    - What level of autonomy do optometrists working in these clinics have?
    - How well do these clinics work?
  - If no
    - Have attempts been made to develop such services?
    - What barriers have there been to developing the role of optometrists delivering glaucoma care?
- Do you/optometrists in your place of work deliver glaucoma related laser procedures?
  - If yes
    - When and how were these services set up?
    - What level of training, accreditation and support do optometrists have?
    - Do these clinics work well?
    - What enablers were there to setting up such services?
    - What barriers were there to setting up such services?
  - If no
    - Why haven’t such services been set up?
    - Are there plans in place to develop such services?
- What do you think patients feel about having an optometrist delivering their glaucoma care?
  - Do you think patients are always aware they are seeing an optometrist and not a doctor?
  - Do you think patients understand what an optometrist is and what their role involves?
  - Are you aware if patients are given written information about the type of clinic they are attending in advance?
  - Do you feel patients feel differently about attending your clinic as opposed to a more traditional glaucoma clinic?
  - Are there any particular aspects of the clinic that patients often compliment you on?
  - Are there any particular aspects of the clinic that patients often complain about?
- How do you see the role of optometrists in glaucoma care develop in the future?
  - How would you like to see your/their role progress?
  - What would you say the enablers will be to progression of the role of optometrists?
  - What do you think are the barriers to progression of the role of optometrists?
  - Do you have any concerns about the expanding role of optometrists delivering care?
- What are the things you like best about working/having optometrists work in the glaucoma clinic?
- What are the things you like least about working/having optometrists work in the glaucoma clinic?
- If you could change one thing about the glaucoma clinic you work in what would it be?
